# Upregulation of an inward rectifying K^+^ channel can rescue slow Ca^2+^ oscillations in K(ATP) channel deficient pancreatic islets

**DOI:** 10.1371/journal.pcbi.1005686

**Published:** 2017-07-27

**Authors:** Vehpi Yildirim, Suryakiran Vadrevu, Benjamin Thompson, Leslie S. Satin, Richard Bertram

**Affiliations:** 1 Department of Mathematics, Florida State University, Tallahassee, FL, United States of America; 2 Brehm Diabetes Center, University of Michigan Medical School, Ann Arbor, MI, United States of America; 3 Department of Mathematics and Programs in Molecular Biophysics and Neuroscience, Florida State University, Tallahassee, FL, United States of America; Oxford, UNITED KINGDOM

## Abstract

Plasma insulin oscillations are known to have physiological importance in the regulation of blood glucose. In insulin-secreting β-cells of pancreatic islets, K(ATP) channels play a key role in regulating glucose-dependent insulin secretion. In addition, they convey oscillations in cellular metabolism to the membrane by sensing adenine nucleotides, and are thus instrumental in mediating pulsatile insulin secretion. Blocking K(ATP) channels pharmacologically depolarizes the β-cell plasma membrane and terminates islet oscillations. Surprisingly, when K(ATP) channels are genetically knocked out, oscillations in islet activity persist, and relatively normal blood glucose levels are maintained. Compensation must therefore occur to overcome the loss of K(ATP) channels in K(ATP) knockout mice. In a companion study, we demonstrated a substantial increase in Kir2.1 protein occurs in β-cells lacking K(ATP) because of SUR1 deletion. In this report, we demonstrate that β-cells of SUR1 null islets have an upregulated inward rectifying K^+^ current that helps to compensate for the loss of K(ATP) channels. This current is likely due to the increased expression of Kir2.1 channels. We used mathematical modeling to determine whether an ionic current having the biophysical characteristics of Kir2.1 is capable of rescuing oscillations that are similar in period to those of wild-type islets. By experimentally testing a key model prediction we suggest that Kir2.1 current upregulation is a likely mechanism for rescuing the oscillations seen in islets from mice deficient in K(ATP) channels.

## Author summary

Pulsatile insulin secretion is important for the proper regulation of blood glucose, and disruption of this pulsatility is a hallmark of type II diabetes. An ion channel was discovered more than three decades ago that conveys the metabolic state of insulin-secreting β-cells to the plasma membrane because it is blocked by ATP and opened by ADP, and thereby controls the activity of these electrically-excitable cells on a rapid time scale according to the prevailing blood glucose level. In addition to setting the appropriate level of insulin secretion, K(ATP) channels play a key role in generating the oscillations in cellular activity that underlie insulin pulsatility. It is therefore surprising that oscillations in activity persist in islets in which the K(ATP) channels are genetically knocked out. In this combined modeling and experimental study, we demonstrate that the role played by K(ATP) current in wild-type β-cells can be taken over by an inward-rectifying K^+^ current which, we show here, is upregulated in β-cells from SUR1 knockout mice. This result helps to resolve a mystery in the field that has remained elusive for more than a decade, since the first studies showing oscillations in SUR1^-/-^ islets.

## Introduction

Insulin is secreted from pancreatic islet β-cells in response to elevated blood glucose. Islet activity is oscillatory, with periods ranging from tens of seconds to several minutes, and this is reflected in the reported periods of pulsatile insulin secretion [1–4]. Plasma insulin oscillations play a physiological role in blood glucose regulation [5–8]. A recent study showed that the action of insulin on the liver to lower plasma glucose is more profound when insulin is delivered to the liver in a pulsatile fashion [9], and earlier studies showed that plasma insulin oscillations are disrupted in type II diabetics and their near relatives [10–12].

At stimulatory levels of glucose β-cells exhibit electrical bursting, and Ca^2+^ that enters the cells during each burst evokes a pulse of insulin secretion [7,13,14]. Several mechanisms have been proposed to explain this bursting electrical activity [15–18]. A recent mathematical model that combines two of these mechanisms can reproduce bursting having a wide range of periods, as seen in experimental studies [19]. One mechanism produces fast oscillations, while the other produces slow oscillations and both can oscillate independently, prompting the name Dual Oscillator Model (DOM). In the DOM, the fast component of bursting results from the negative feedback of Ca^2+^ on the membrane potential via Ca^2+^-activated K^+^ channels and, indirectly, via K(ATP) channel activation. The slow component, in contrast, is due to oscillations in glycolysis that occur as the result of actions of the allosteric enzyme phosphofructokinase (PFK)[20,21]. The subsequent oscillatory ATP production acts through ATP-sensitive K^+^ channels (K(ATP) channels) to produce oscillations in K(ATP) current, which turns the bursts of electrical activity on and off [22,23].

K(ATP) channels play a crucial role coupling cell metabolism to membrane potential. These channels are comprised of four inwardly rectifying K^+^ channel subunits (Kir6.2) and four sulfonylurea receptor subunits (SUR1) arranged in an octomeric array (for review see [24]). A mutation in the genes coding for either subunit prevents K(ATP) channels from being trafficked normally to the plasma membrane or alters their sensitivity to adenine nucleotides, leading to persistent hyperinsulinemic hypoglycemia of infancy (PHHI) in humans, a condition characterized by high insulin secretion that occurs even when blood glucose is low [25–27]. High secretion results from the permanent depolarization of the β-cell membrane that is due to the lack of normally hyperpolarizing K(ATP) current. Surprisingly, in SUR1 homozygous knockout mice (SUR1^-/-^ mice), lacking K(ATP) channels, islets typically still exhibit electrical bursting (although the glucose sensitivity of bursting in these islets is largely abrogated), and blood glucose levels are relatively normal unless the animals are metabolically stressed [28,29]. Similarly, islets from Kir6.2 knockout mice exhibit slow Ca^2+^ oscillations, similar to those observed in wild-type islets which are known to be due to bursting electrical activity [30]. In these mice, compensation must therefore occur to overcome the loss of the large hyperpolarizing K(ATP) current. Indeed, when the K(ATP) channels of wild type islets are acutely blocked by sulfonylurea drugs, β-cells spike continuously from a sustained depolarized level [31–33]. We hypothesized that such compensation could be achieved through the upregulation of another hyperpolarizing K^+^ channel that impersonates K(ATP) channels in sensing cellular metabolism [34]. In a companion study (Vadrevu et al, manuscript in preparation), we demonstrated that the upregulation of Kir2.1 channel protein in islets from SUR1^-/-^ mice (KO islets) could mediate this compensation. In the current report, we demonstrate that SUR1 KO islets exhibit sustained Ca^2+^ oscillations at stimulatory levels of glucose, and that the amount of inward rectifying K^+^ current is increased in these K(ATP) channel KO cells. Using mathematical modeling, we explored the functional role of this current on the electrical activity of islet β-cells when K(ATP) channels are absent. In particular, we investigated whether this inward-rectifying K^+^ current has the ability to rescue normal electrical bursting pattern in β-cells of SUR1^-/-^ mouse islets.

Kir2.1 channels conduct large inward currents at voltages below the K^+^ Nernst potential (*V*_*K*_) and smaller outward currents at voltages above *V*_*K*_. This diode-like property, or inward rectification, is caused by blockade of the channels by intracellular ions and polyamines when the cell membrane is depolarized [35–37]. Kir2.1 channels also contain consensus sites for phosphorylation by protein kinase A (PKA) and studies show that PKA potentiates Kir2.1 current [38–40]. One study shows that a phosphatase inhibitor can prevent rundown of the Kir2.1 current that is activated by PKA, which indicates activation of the channels is regulated by protein phosphorylation [41]. Since PKA activity is cAMP-dependent, changes in the cAMP concentration in the β-cell can in principle regulate Kir2.1 channel activity. Recent studies employing FRET-based sensors and TIRF microscopy showed that glucose induces cAMP oscillations in mouse β-cells [42,43], which may be accounted for by oscillations in metabolism [44]. It is therefore possible that, in KO cells, metabolic oscillations drive cAMP oscillations which in turn drive oscillations in Kir2.1 current, and this replaces oscillations in K(ATP) current as the mechanism for bursting electrical activity. We illustrate how this works with the model, and make predictions that are subsequently confirmed experimentally and thereby support the hypothesis that Kir2.1 channel upregulation is a feasible mechanism which can rescue electrical bursting in SUR1^-/-^ mouse islets lacking K(ATP) channels.

## Materials and methods

### Ethics statement

The animal protocol used was in accordance with the guidelines of the University of Michigan Institutional Animal Care and Use Committee (IACUC).

### Islet preparation

Pancreatic islets were isolated from 3–4 month old male Swiss-Webster mice as in [45]. Islets were hand picked into fresh Kreb’s solution and then transferred to culture dishes containing RPMI-1640 supplemented with 10% FBS, glutamine and penicillin-streptomycin. Islets were cultured overnight at 37°C in an incubator. Electrophysiological recordings were made from islets cultured for 72 hours or less.

### Electrophysiology

Patch electrodes were pulled (P-97, Sutter Instrument Co., Novato, CA) from borosilicate glass capillaries (Warner Instrument Inc., Hamden, CT) and had resistances of 8–10 M-ohm when filled with an internal buffer containing (in mM): 28.4 K_2_SO_4_, 63.7 KCl, 11.8 NaCl, 1 MgCl_2_, 20.8 HEPES and 0.5 EGTA at pH7.2. The electrodes were then backfilled with the same solution but containing amphotericin B at 0.3 mg/ml to allow membrane perforation. Islets were transferred from culture dishes into a 0.5 ml recording chamber held at 32–34°C. Islets were visualized using an inverted epifluorescence microscope (Olympus IX50, Tokyo, Japan). Pipette seals obtained were > 1 G-ohms. Recordings were made using an extracellular solution containing (in mM): 135 NaCl, 2.5 CaCl_2_, 4.8 KCl, 1.2 MgCl_2_, 20 HEPES, and 11.1 glucose.

### Voltage ramps

After the establishment of a perforated patch, cells were voltage-clamped to a holding potential of -60 mV, and a 2-second voltage ramp from -120 to -50 mV was applied, as in [32]. Evoked currents were digitized at 10 kHz after filtering at 2.9 kHz. The protocols were generated using Patchmaster software (v2x32; HEKA Instruments).

### Fura-2 imaging of cytosolic Ca^2+^ and pharmacological treatments

Pancreatic islets were cultured overnight in RPMI medium containing 5 mM glucose and on the day of experiments were transferred to fresh media containing 2.5 μM Fura-PE2-AM for 30 min. Following incubation, islets were loaded into a glass-bottomed chamber mounted onto the microscope stage. The chamber was perfused at 0.3 mL/min with 11 mM glucose solution and the ambient temperature was maintained at 33°C using inline solution and chamber heaters (Warner Instruments). Excitation was provided by a TILL Polychrome V monochromator (TILL Scientific, Germany) with light output set to 10% maximum. Excitation (x) or emission (m) filters (ET type; Chroma Technology, Bellows Falls, VT) were used in combination with an FF444/521/608-Di01 dichroic (Semrock, Lake Forest, IL) as follows: Fura-2, 340/10x and 380/10x, 535/30m (R340x/380x – 535m); A single region of interest was used to quantify the average response of each islet using MetaMorph software (Molecular Devices). In one set of experiments, after three oscillations were recorded, the solution was switched to a solution containing 11 mM glucose with thapsigargin (1 μM). In another set of experiments, the solution was switched to one containing 11 mM glucose and 8-Bromoadenosine 3’,5’-cyclic monophosphate (8-Br-cAMP) (50 μM).

### Modeling

**Fig 1:**
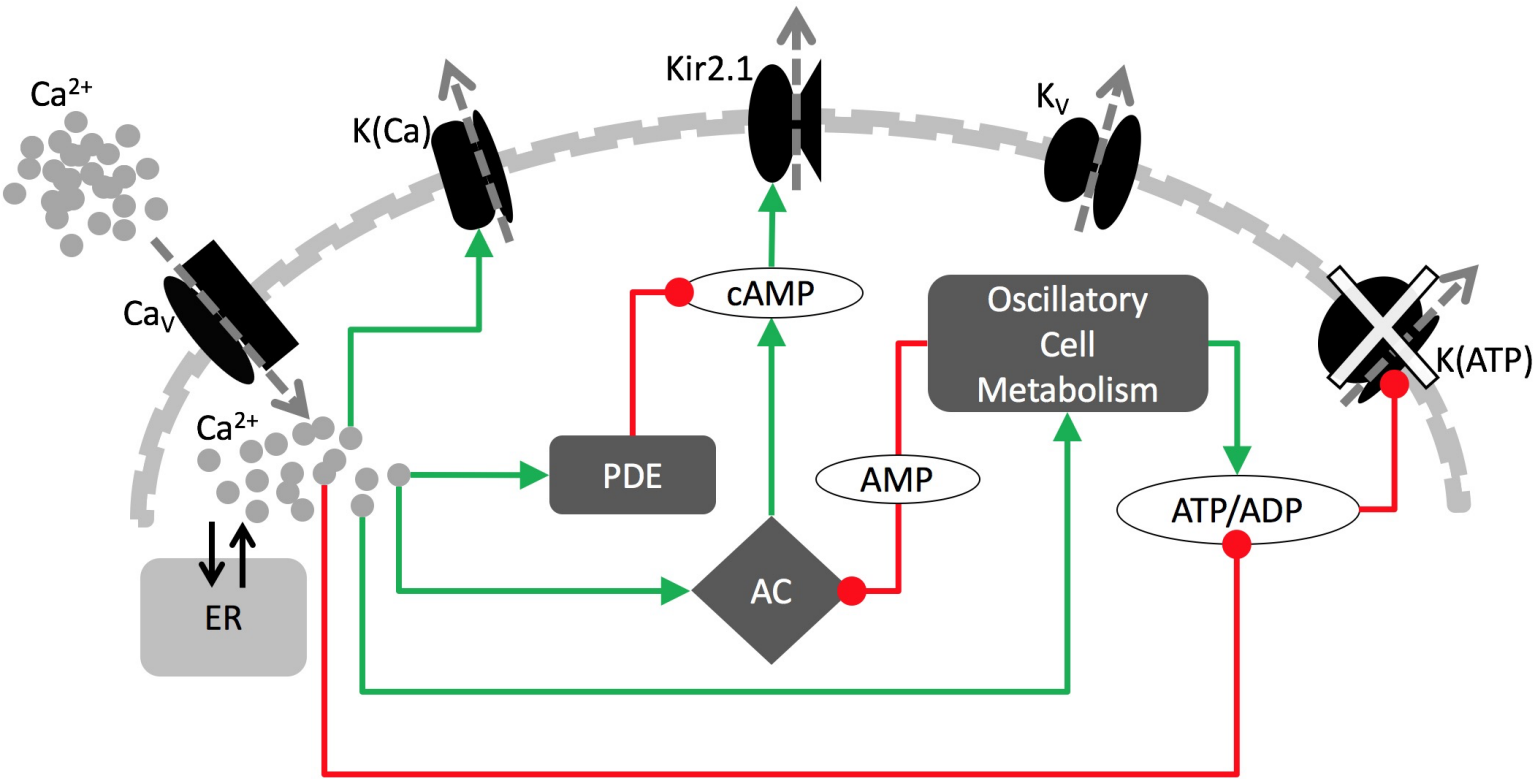
The key components of the model. Green arrows are for stimulatory and red circles are for inhibitory pathways. In the wild-type cells, bursting is paced by metabolic oscillations acting on K(ATP) channels. In the KO cells, genetic disruption of K(ATP) channels leads to increased Kir2.1 current, which now drives bursting.

We used an 8-variable model consisting of ordinary differential equations, illustrated in Fig 1. This Dual Oscillator Model (DOM) has electrical, Ca^2+^, and metabolic components [23,46]. We focus our description on elements of the model that are most important for this study, but all equations and tables of parameter values are given in Supporting Information. (The computer codes, using the CVODE solver implemented in XPPAUT, can be downloaded as freeware from www.math.fsu.edu/~bertram/software/islet.) In the DOM, the fast oscillatory component is based on negative Ca^2+^ feedback onto the membrane potential through Ca^2+^-sensitive K^+^ current (I_K(Ca)_). This mechanism can drive fast bursting. The second oscillatory component is due to metabolic oscillations, which result from the activity of the allosteric enzyme phosphofructokinase (PFK). In the process of glycolysis, PFK catalyzes the phosphorylation of fructose 6-phosphate (F6P) to fructose 1,6-bisphosphate (FBP). The activity of PFK is increased by its product FBP, so that increased FBP increases the reaction rate and causes a sharp rise in FBP. This eventually depletes the substrate of the reaction, F6P, and turns off flux through PFK, resulting in a reduction in FBP. This allows the substrate, F6P, to recover and the cycle to restart. Oscillatory FBP levels in turn cause oscillations in pyruvate, the end product of glycolysis and the substrate for mitochondrial respiration. The oscillatory glycolytic input results in oscillatory levels of the nucleotide concentrations (ATP, ADP and AMP). The membrane potential is then affected through the action of ATP and ADP on K(ATP) channels, which can drive slow bursting in the model.

Equations for the dynamics of cAMP were recently added to an earlier version of the DOM [44] and it was shown that this version was capable of producing cAMP oscillations in model β-cells. We employed these equations, where the cAMP concentration is determined by the difference between its production by adenylyl cyclase (*V*_*AC*_) and degradation by phosphodiesterases (*V*_*PDE*_):

$$\frac{dcAMP}{dt}=V_{AC}-V_{PDE}$$ (1)

where,

$$V_{AC}={\bar{v}}_{AC}\left(\alpha_{AC}+\beta_{AC}\frac{c^{3}}{c^{3}+K_{ACca}^{3}}\right)\left(\beta_{amp}\frac{K_{amp}^{2}}{{AMP}_{c}^{2}+K_{amp}^{2}}\right)$$ (2)

$$V_{PDE}={\bar{v}}_{PDE}\left(\alpha_{PDE}+\beta_{PDE}\frac{c^{3}}{c^{3}+K_{PDEca}^{3}}\right)\frac{cAMP}{cAMP+K_{PDEcamp}}$$ (3)

where *c* is the cytosolic free Ca^2+^ concentration, which stimulates both AC and PDE. Cytosolic AMP (*AMP*_*c*_) inhibits AC and thus the production of cAMP [47–49]. We modified the *V*_*AC*_ equation from the original model to incorporate a higher-order dependence on AMP. In the model, slow cAMP oscillations are the result of AMP oscillations and the accompanying Ca^2+^ oscillations, which are both the product of glycolytic oscillations. The details of the cAMP dynamics are given in [44].

In the DOM, the rate of change of the membrane potential of a wild type β-cell is given by a conductance-based Hodgkin-Huxley type equation:

$$\frac{dV}{dt}=-(I_{K}+I_{Ca}+I_{K(Ca)}+I_{K(ATP)})/C_{m}$$ (4)

where, *C*_*m*_ is the membrane capacitance, *I*_*K*_ is the delayed rectifier K^+^ current, *I*_*Ca*_ is a voltage-sensitive Ca^2+^ current, *I*_*K(Ca)*_ is a Ca^2+^-sensitive K^+^ current and *I*_*K(ATP)*_ is an ATP-sensitive K^+^ current. The rate of change of the free cytosolic Ca^2+^ concentration is:

$$\frac{dc}{dt}=f_{cyt}\left(\overset{J_{mem}}{\overbrace{{-\alpha I}_{Ca}-k_{pmca}c}}+\overset{J_{ER}}{\overbrace{k_{leak}(c_{er}-c)-k_{SERCA}c}}\right)$$ (5)

where terms labeled by *J*_*mem*_ and *J*_*ER*_ represent the Ca^2+^ flux across the plasma membrane and net flux out of the endoplasmic reticulum (ER), respectively. Here, *f*_*cyt*_ is the fraction of free to total cytosolic Ca^2+^, α converts current to flux, *k*_*pmca*_ is the Ca^2+^ pumping rate across the plasma membrane, *k*_*leak*_ is the rate of the Ca^2+^ leak from the ER and *k*_*SERCA*_ is the Ca^2+^ pumping rate into the ER. The ER Ca^2+^ concentration (*c*_*er*_) is also dynamic and given by:

$$\frac{{dc}_{er}}{dt}=-f_{er}V_{cte}(k_{leak}(c_{er}-c)-k_{SERCA}c)$$ (6)

where *f*_*er*_ is the ratio of the free Ca^2+^ in the ER and *V*_*cte*_ is the ratio of the volume of the cytosol to the volume of the ER compartment. The equation for the Ca^2+^-sensitive K^+^ current (*I*_*K(Ca)*_) is,

$$I_{K(Ca)}=g_{K(Ca)}\omega (V-V_{K})$$ (7)

where, *g*_*K(Ca)*_ is the maximal conductance of the current, and ω is the following Ca^2+^-dependent activation function,

$$\omega =\frac{c^{2}}{c^{2}+K_{c}^{2}}$$ (8)

where *K*_*c*_ is the affinity constant.

In the KO-cells lacking K(ATP) channels there is no *I*_*K(ATP)*_ present. In the model KO-cells, K(ATP) current is replaced by the following Kir2.1-mediated inward-rectifying K^+^ current:

$$I_{Kir}=g_{Kir}k_{\infty }c_{\infty }(V-V_{K}).$$ (9)

**Fig 2:**
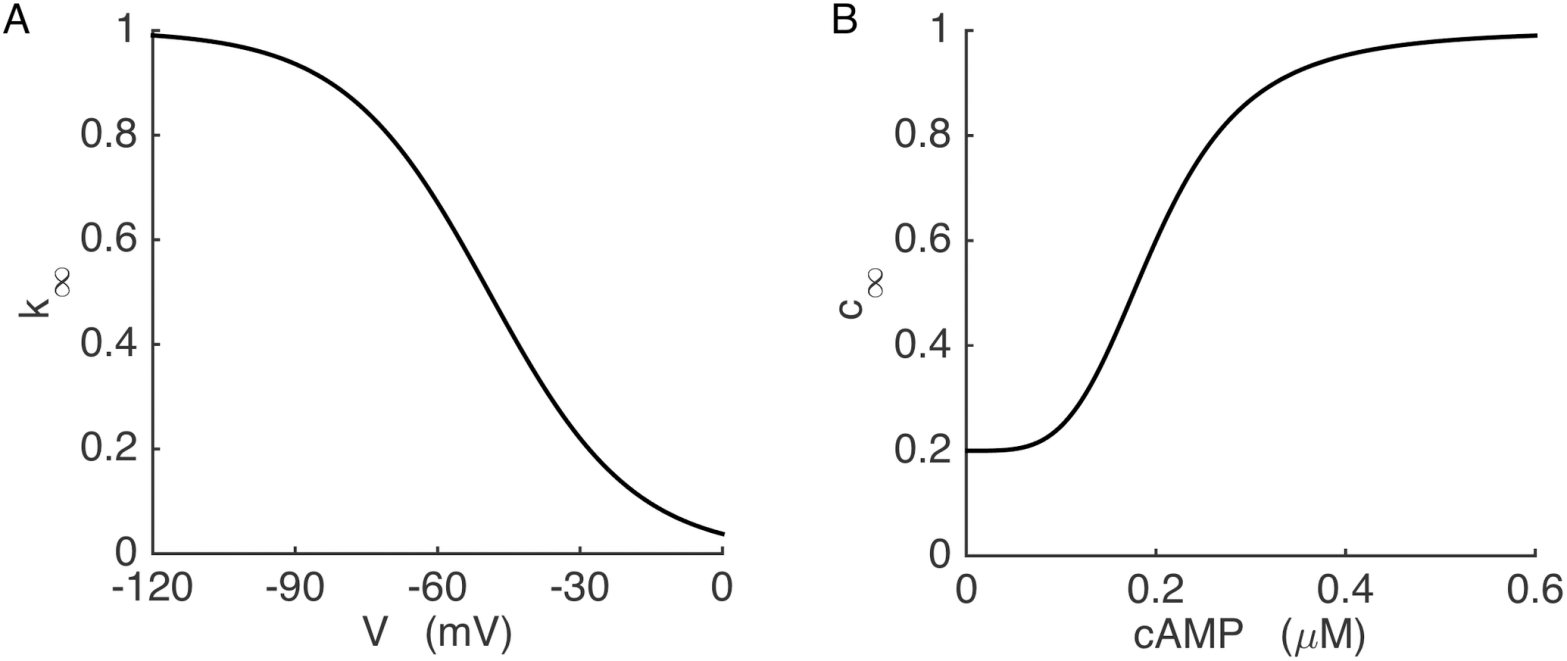
The Kir2.1 channel conductance depends on voltage and the cAMP concentration. (A) Voltage-dependent blockade of the Kir2.1 current. (B) cAMP-dependent activation of the Kir2.1 current.

Here *g*_*Kir*_ is the maximal Kir2.1 channel conductance, *k∞* is the voltage-dependent block of the channel by polyamines which is the cause of the inward rectification, and *c∞* is the cAMP-dependent activation of the channels. We use a Boltzmann function to describe *k∞*:

$$k_{\infty }=\frac{1}{1+exp(\frac{V-V_{kir}}{s_{kir}})}$$ (10)

where *V*_*Kir*_ is the half activation potential and *s*_*Kir*_ is the slope factor that determines the sensitivity to the voltage. The resulting voltage-dependent *k∞* curve is shown in Fig 2A and is parameterized according to [50]. Kir2.1 current has both cAMP dependent and independent components [38], which are incorporated into the activation function *c*_*∞*_ as follows:

$$c_{\infty }=\alpha_{camp}+\beta_{camp}\frac{{cAMP}^{4}}{{cAMP}^{4}+K_{camp}^{4}}$$ (11)

where *α*_*camp*_ is the cAMP independent component, and the cAMP dependency of the current is described by the second term. The *c*_*∞*_ function is illustrated in Fig 2B.

## Results

### An inward-rectifying K^+^ current is upregulated in SUR1^-/-^ pancreatic β-cells

**Fig 3:**
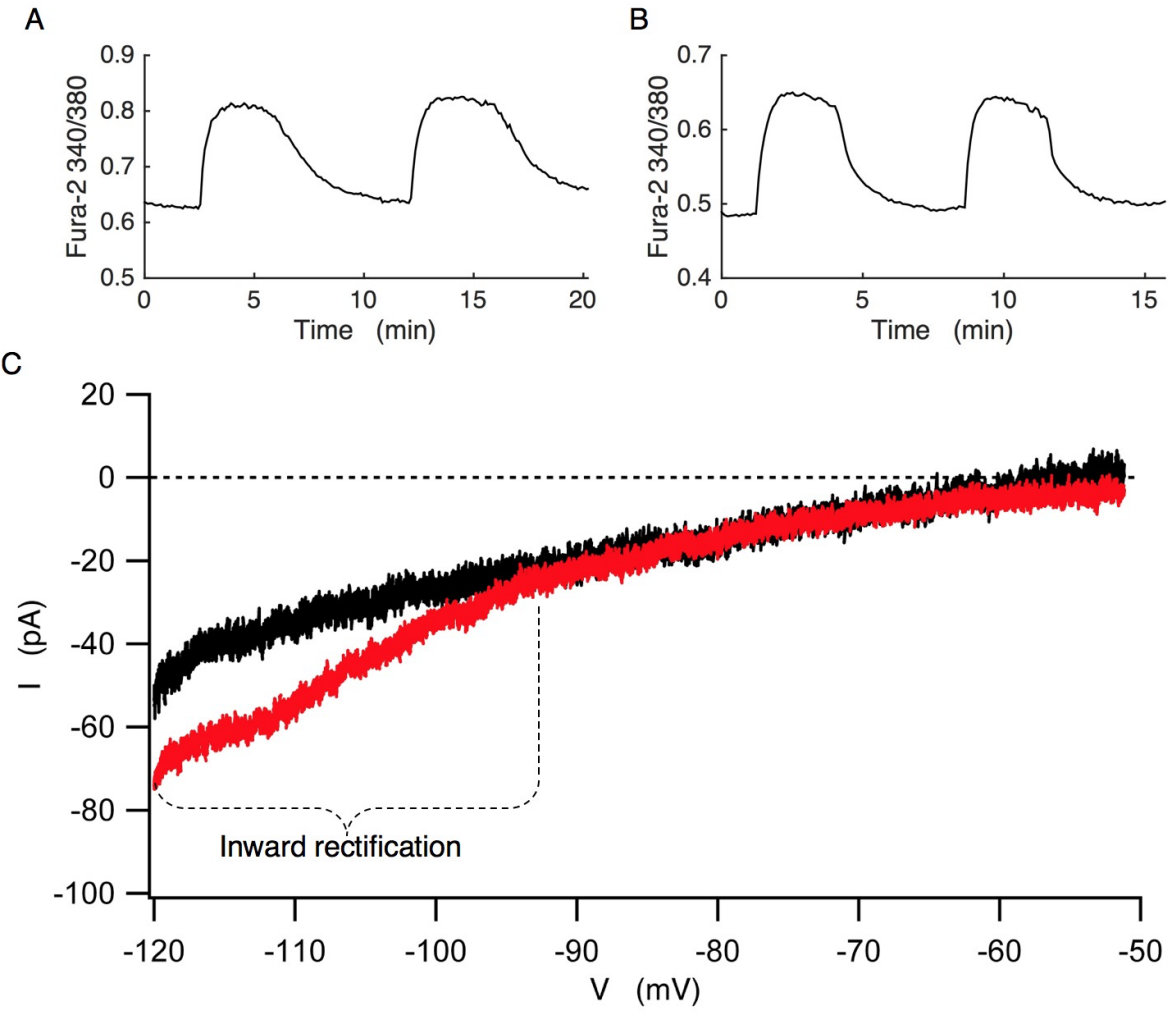
Fura-2 Ca^2+^ measurements from wild-type (A) and SUR1^-/-^ islets (B) at 11 mM glucose. The change in Ca^2+^ is expressed as the Fura-2 340/380 ratio. (C) Comparison of I-V curves from wild-type (black) and SUR1^-/-^ (red) β-cells. The wild-type recording is representative of n = 6 islets isolated from 4 mice. The SUR1^-/-^ recording is representative of n = 8 islets isolated from 5 mice. The SUR1^-/-^ islets exhibited significant inward rectification at more negative potentials compared to cells from wild-type islets.

Ca^2+^ and membrane potential oscillations in SUR1^-/-^ islets lacking functional K(ATP) channels were reported previously [28,51]. Our fura-2 Ca^2+^ measurements verified that slow cytosolic Ca^2+^ oscillations persisted in both wild-type (Fig 3A) and KO-islets (Fig 3B) perfused with 11 mM glucose. These data show that our SUR1^-/-^ islets recapitulate the Ca^2+^ oscillations observed in [28,51].

We recently identified an increase in Kir2.1 channel protein in islets isolated from SUR1^-/-^ mice (Vadrevu et al, manuscript in preparation). To verify the electrophysiological functionality of these channels in the β-cell membrane of KO islets, we measured current-voltage relations of wild-type and KO cells using the perforated patch clamp technique in peripheral islet β-cells. Fig 3C shows current recordings elicited by voltage ramp commands from -120 mV to -50 mV (see Materials and Methods) applied to wild-type islets (black) and K(ATP) KO islets (red).

In wild-type islets, the current-voltage relation is largely linear beyond about -110 mV (Fig 3C, black) (n = 6 islets from 4 mice), while in the KO cells the evoked current was more nonlinear, exhibiting inward rectification (Fig 3C, red). The strong inward rectification is likely due to current from the upregulated Kir2.1 inward-rectifying K^+^ channels that we report in a companion study (Vadrevu et al, manuscript in preparation), supporting a functional role for the upregulated Kir2.1 channel protein.

### The model demonstrates that Kir2.1 channel upregulation can rescue bursting in SUR^-/-^ islets

**Fig 4:**
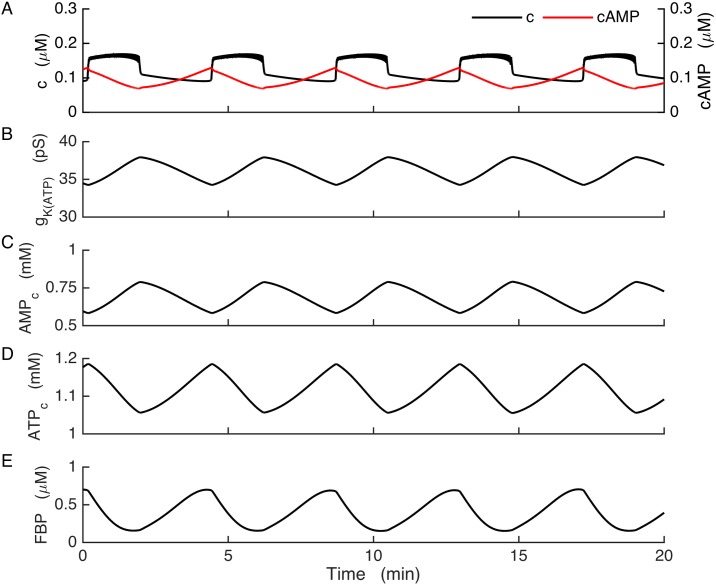
Bursting in wild-type model cells. Slow glycolytic oscillations drive bursting through actions on the K(ATP) current. (A) cAMP declines at the start of each Ca^2+^ plateau. (B) K(ATP) channel conductance. (C-D) Adenine nucleotide concentrations in the cytosol. (E) Slow glycolytic oscillations are reflected in the FBP time course.

Fig 4 illustrates slow bursting produced by the model for the case of wild-type cells. The oscillations in the free Ca^2+^ concentration observed here (Fig 4A) result from the bursting electrical activity described earlier. The burst timing in this case is controlled by the slow glycolytic oscillations, which are reflected by the FBP time course as shown (Fig 4E). FBP oscillations in turn cause oscillations in downstream metabolic components, including cytosolic AMP and ATP (Fig 4C and 4D). The conductance of K(ATP) channels (*g*_*K(ATP)*_) is dependent on ADP and ATP levels, and oscillations in the concentrations of these nucleotides cause K(ATP) conductance (Fig 4B) and concomitantly K(ATP) current to oscillate and drive slow busting. The slow cAMP oscillations are modulated by Ca^2+^ and AMP, but in the model of the wild-type β-cells cAMP has no impact on the cell’s electrical activity.

If the key K(ATP) channels are removed, the model cell spikes continuously, as is seen experimentally when a K(ATP) channel blocker like tolbutamide is applied to a wild-type islet [31–33]. The upregulated Kir2.1 conductance shown in Fig 3C would be expected to also provide hyperpolarizing current, but can it rescue the bursting oscillations that are normally driven by K(ATP) current? To answer this, we replaced K(ATP) current in the model with Kir2.1 current to simulate the case for KO cells. The properties of this model current are discussed in Materials and Methods and are shown in Fig 2. A key feature of the Kir2.1 channels is their activation by cAMP [38–40].

**Fig 5:**
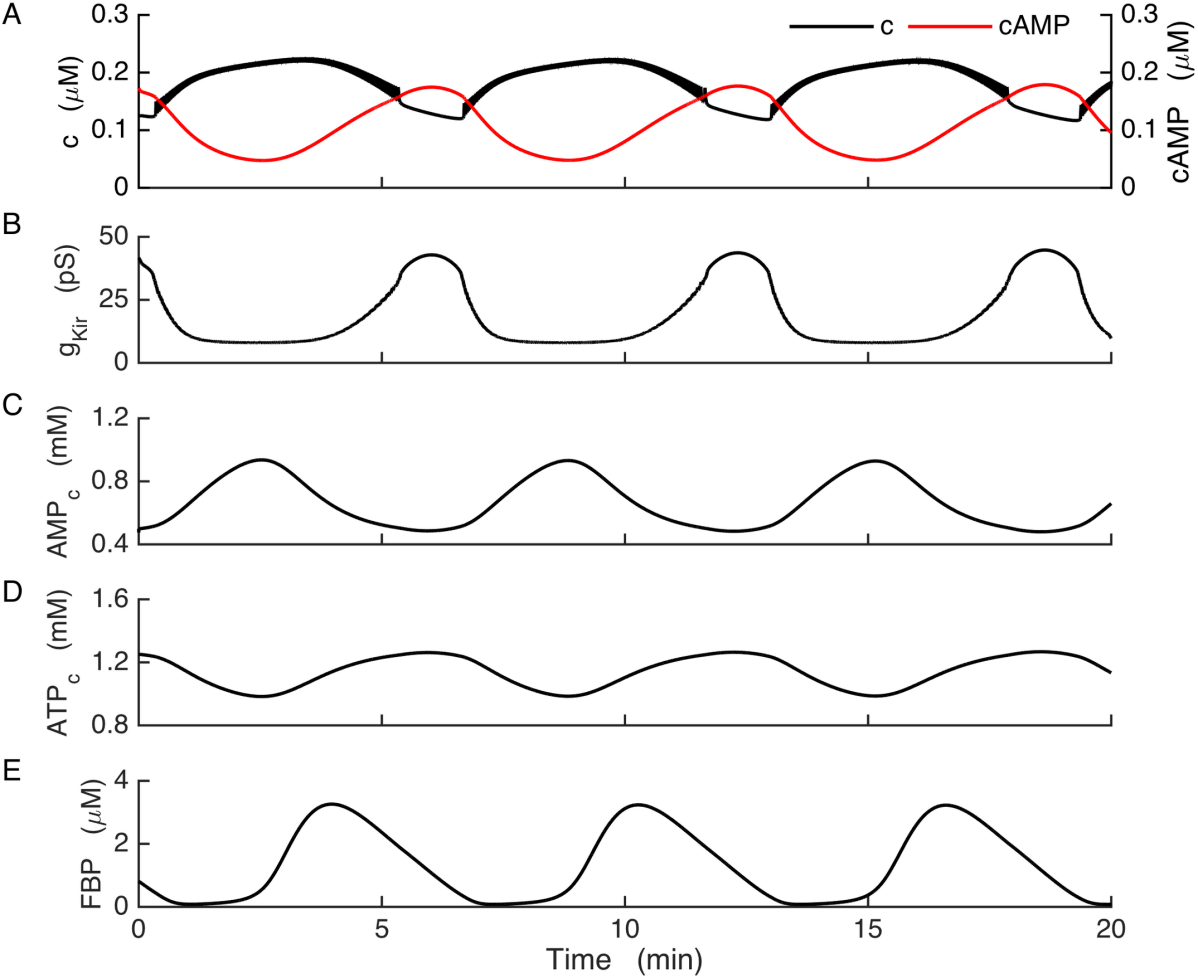
Bursting in the model KO cells, where K(ATP) current is replaced with Kir2.1 current. Glycolytic oscillations drive bursting through a cAMP-dependent pathway. (A) Ca^2+^ and cAMP concentrations oscillate in anti-phase. (B) Conductance of the Kir2.1 current, time averaged over a window of 6 s to remove fast variations and highlight the cAMP-dependent slow dynamics. (C) *AMP*_*c*_ oscillations contribute to the production of cAMP oscillations. (D) *ATP*_*c*_ oscillates due to oscillations in glycolysis. (E) FBP is the product of the PFK enzyme that is responsible for glycolytic oscillations. For this simulation, the glucokinase reaction rate was increased from 0.09 μM/ms to 0.14 μM/ms and *k*_*FBP*_ was increased from 0.8 to 0.95.

In Fig 5 we show that if Kir2.1 is sufficiently up-regulated, it can rescue slow bursting in model cells lacking K(ATP). In the model of the KO condition, slow glycolytic oscillations now drive slow *AMP*_*c*_ oscillations (Fig 5C) that cause the cAMP concentration to oscillate (Fig 5A, red). cAMP in turn activates the Kir2.1 channels and results in oscillations in the Kir2.1 conductance (Fig 5B). This causes the membrane potential to switch between the active and silent phases, which drives bursting and Ca^2+^ oscillations as in the wild-type case (Fig 5A, black). The shape of the burst is largely determined by the details of the V and cAMP dependence of the Kir2.1 channels, which in our model is calibrated by data from a human isoform of the channel expressed in human embryonic kidney cells. Differences of channel properties between mouse and human would change the shape of the burst, but not the burst mechanism (unless channel differences were drastic). A robust property of the burst mechanism is that the cAMP concentration peaks during the silent phase in the KO model cells, unlike the wild-type model cells where cAMP peaks at the beginning of the active phase. This peak in cAMP is reflected in the Kir2.1 conductance. Fig 5B shows the moving average of this conductance, where averaging is done over a window of 6 s to filter out fast V-dependent changes. Like cAMP, the Kir2.1 conductance peaks during the silent phase, and the subsequent decline in this conductance starts the next burst. Although the ATP concentration also oscillates (Fig 5D), it does not affect the membrane potential in this case since there are no K(ATP) channels to sense changes in nucleotides.

**Fig 6:**
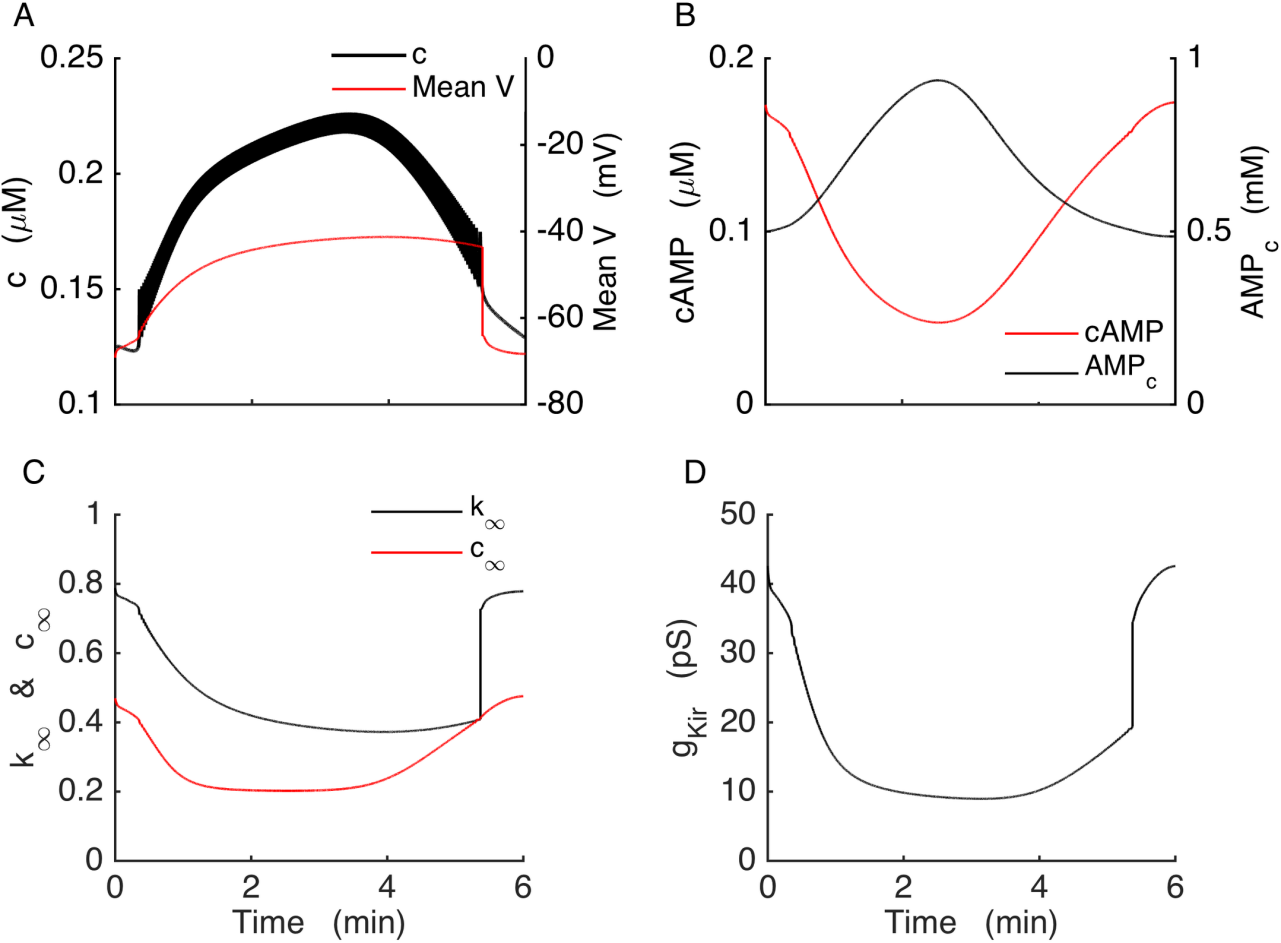
In the model KO cells, bursting is driven by the Kir2.1 current, which is regulated by voltage and the cAMP concentration. (A) Mean *V* and *c* during a burst. Voltage is averaged over each spike. (B) The cAMP and cytosolic AMP concentrations. (C) Dynamics of the Kir2.1 channel activation (*c*_∞_) and inactivation (*k*_∞_). (D) Kir2.1 conductance during a burst.

In the wild-type model cells, cAMP had no effect on membrane potential or any other components of the model. However, in the model we made of the KO cells, cAMP, acting through Kir2.1 channels, is now the key to slow bursting. To further understand how this occurs, a slow burst is shown in more detail in Fig 6. In this figure, voltage is averaged over the duration of each spike to illustrate mean voltage (Fig 6A, red). This allows us to focus on the slower burst waveform. The figure begins with the system in the silent phase, where Kir2.1 conductance is high (Fig 6D) due to elevated cAMP concentration (Fig 6B, red) and a relatively hyperpolarized voltage (Fig 6A, red). As glycolytic activity declines near the end of the silent phase *AMP*_*c*_ slowly increases (Fig 6B, black). This, in turn, reduces the cAMP concentration by inhibiting adenylyl cyclase, thereby reducing Kir2.1 channel activation (Fig 6C, red). The resulting decline in Kir2.1 conductance initiates an active phase of electrical activity, further reducing Kir2.1 conductance due to voltage-dependent channel blockade as the cell depolarizes (Fig 6C, black). Cytosolic Ca^2+^ now increases due to Ca^2+^ influx through voltage-dependent Ca^2+^ channels and this activates Ca^2+^-ATPase pumps through ATP hydrolysis, further increasing the *AMP*_*c*_. This causes cAMP to decline rapidly. By the middle of the active phase AMP reaches its peak and then starts to decline. This decline, despite the continued rise in *c*, is due to the upstroke of the glycolytic oscillator, which facilitates the production of ATP at the expense of ADP and AMP. Decreased *AMP*_*c*_ disinhibits adenylyl cyclase and cAMP again starts to increase. The cytosolic Ca^2+^ concentration starts to decrease only after cAMP is elevated enough to significantly activate Kir2.1 current (Fig 6C, red), eventually terminating the active phase.

### Oscillations are terminated by 8-Br-cAMP

The KO model relies on the action of cAMP oscillations on Kir2.1 channels to drive electrical bursting and Ca^2+^ oscillations in the SUR1^-/-^ islets. If cAMP is tonically elevated, then the subsequent tonic activation of Kir2.1 should hyperpolarize the islet, terminating electrical bursting and Ca^2+^ oscillations, and bringing the intracellular Ca^2+^ concentration to a low level. We performed this manipulation by adding 8-Bromoadenosine 3’,5’-cyclic monophosphate (8-Br-cAMP) to wild-type and SUR1^-/-^ islets. This is a membrane permeant brominated derivative of cAMP that is resistant to degradation by cAMP phosphodiesterase, and is thus long lasting.

**Fig 7:**
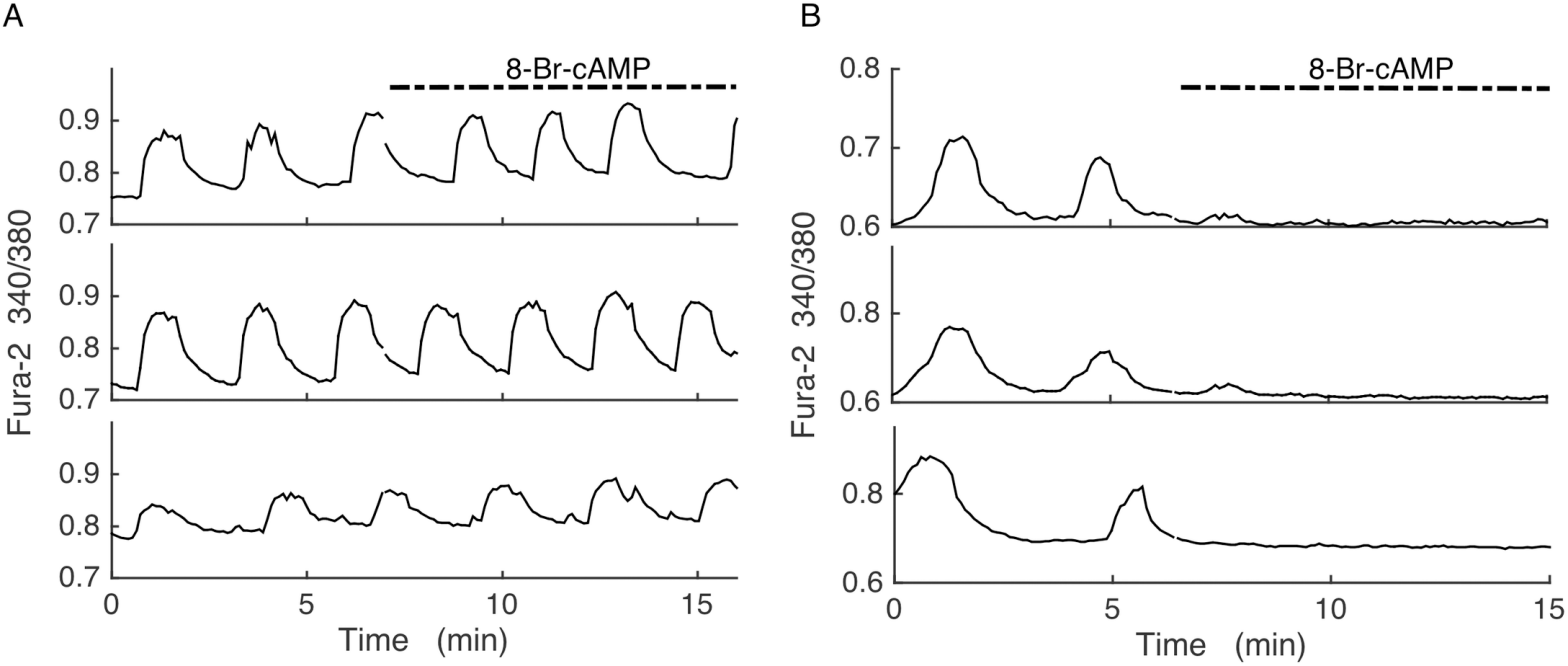
Fura-2 Ca^2+^ measurements of islets in 11 mM glucose and, as indicated, 50 μM of the membrane permeable 8-Br-cAMP. (A) Ca^2+^ oscillations in wild-type islets persist with little or no change upon application of 8-Br-cAMP. Representative of 10 islets. (B) Ca^2+^ oscillations in SUR1^-/-^ islets are terminated by 8-Br-cAMP, and Ca^2+^ is at a low level. Representative of 9 islets.

Application of 8-Br-cAMP (50 μM) to wild-type islets (N = 10) had little or no effect on Ca^2+^ oscillations, as shown in three representative islets (Fig 7A). In contrast, the same concentration applied to SUR1^-/-^ islets terminated Ca^2+^ oscillations in all islets tested (N = 9), reducing the intracellular Ca^2+^ level to what is expected from a hyperpolarized islet (Fig 7B). This is consistent with the hypothesis that cAMP activates Kir2.1 channels, and that oscillations in cAMP drive oscillations in Ca^2+^ in SUR1^-/-^ islets, but not wild-type islets.

### Fast/slow analysis of the Kir2.1 model

**Fig 8:**
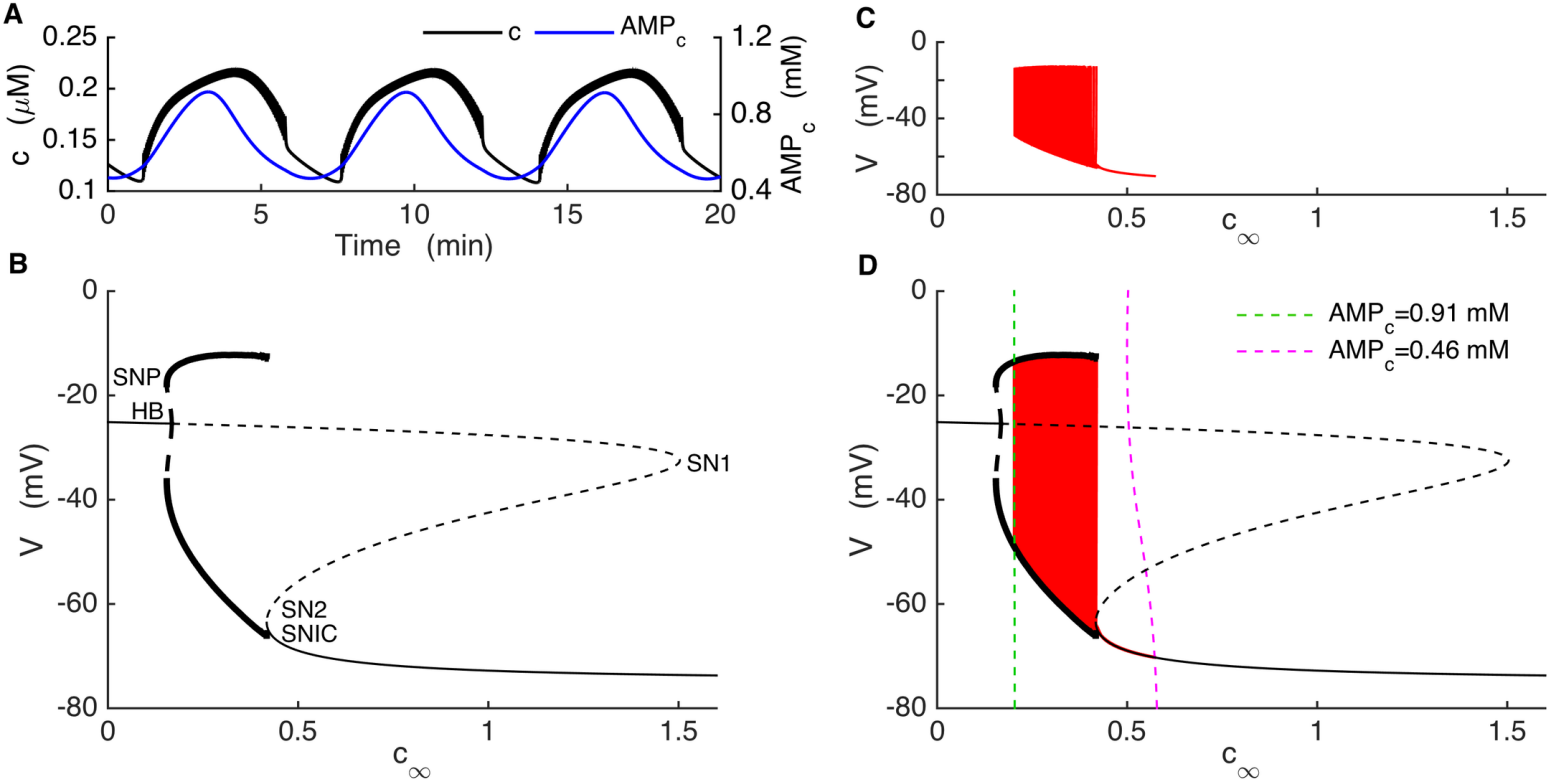
Glycolytic oscillations drive bursting in the model KO cell. (A) *c* (black) oscillates reflecting bursting electrical activity, while *AMP*_*c*_ oscillates (blue) reflecting glycolytic oscillations. (B) Bifurcation diagram of the fast subsystem, with *c*_*∞*_ as bifurcation parameter. HB = Hopf bifurcation, SN = saddle-node bifurcation, SNIC = saddle-node on invariant circle bifurcation. Solid and dashed curves represent stable and unstable steady states, respectively, while bold solid and bold dashed curves represent stable and unstable limit cycles, respectively. (C) The burst trajectory projected onto the *c*_*∞*_-V plane. (D) Fast/slow analysis of bursting, with the burst trajectory (red) and *c*_*∞*_ curve superimposed onto the fast-subsystem bifurcation diagram. The *c*_*∞*_ curve is shown for *AMP*_*c*_ at its minimum (dashed magenta) and maximum (dashed green) during a burst.

To better understand the dynamics of the bursting mechanism, and to help facilitate the design of new experiments, we performed a fast/slow analysis of the Kir2.1 model. Fast/slow analysis separates system variables into component fast and slow subsystems based on their respective time scales [52]. The slow variables are almost constant on the time scale of changes in the fast variables. Therefore, these variables can be treated as slowly-varying parameters of the fast subsystem. In our model, the fast variables are voltage (*V*), the activation variable for voltage-gated K^+^ current (*n*) and cytosolic Ca^2+^ (*c*). The variables that change on much slower time scales are fructose 6-phosphate (*F6P*), fructose 1,6-bisphosphate (*FBP*), *ATP*_*c*_, *AMP*_*c*_, *cAMP* and the Ca^2+^ concentration of the ER (*c*_*er*_). For comparison, Fig 8A shows a fast variable (*c*) shown together with a slow variable *AMP*_*c*_. At the start of a burst active phase *c* immediately jumps to a plateau and exhibits small oscillations due to the voltage spikes, and jumps down at the end of the active phase. In contrast, *AMP*_*c*_ exhibits a slow rise and fall, with a peak near the middle of the active phase. We start the fast/slow analysis by setting *c*_*er*_ to its mean value, since it is not a part of the primary oscillatory mechanism. The slow variables other than *c*_*er*_ interact according to the following scheme:

F6P→FBP→ATP→AMP→cAMP

where only cAMP directly affects the fast subsystem, through the cAMP-dependent activation variable of *I*_*Kir*_ (*c*_*∞*_). We first generate a bifurcation diagram of the fast subsystem with *c*_*∞*_ as the bifurcation parameter (Fig 8B), since the curve is simpler than that obtained using cAMP itself as the bifurcation parameter. For small values of *c*_*∞*_ the system is at a depolarized steady state, since the Kir2.1 current is largely turned off. These stable steady states make up the initial segment of the upper branch of the z-shaped curve (solid line), which we refer to as the z-curve. As *c*_*∞*_ is increased two branches of periodic solutions, one stable (bold solid curve) and one unstable (bold dashed curve), emerge at a saddle node of periodics (SNP) bifurcation. The branch of unstable limit cycles is created at a subcritical Hopf Bifurcation (HB), at which point the branch of stable steady states becomes unstable (dashed curve). The branch of unstable steady states turns at a saddle-node bifurcation (SN1), forming the middle branch of the z-curve. This branch turns at another saddle-node bifurcation (SN2) and forms the stable lower branch of the z-curve. The stable branch of periodic solutions reflects tonic spiking, and the minimum and maximum voltage values during a spike are shown as two separate curves. This branch terminates at the left knee of the z-curve at a saddle-node on invariant circle (SNIC) bifurcation.

The burst trajectory is shown projected into the *c*_*∞*_*-V* plane in Fig 8C. The left portion of the trajectory reflects the active phase of the burst when the model cell is spiking. When the cell enters the silent phase *c*_*∞*_ first increases and then decreases to start a new active phase. This is the right portion of the trajectory. The burst trajectory is superimposed onto the z-curve in Fig 8D, along the *c*_*∞*_ curve (Eq 11). This curve depends on the cAMP concentration, which has the following steady state function:

$${cAMP}_{ss}=\frac{k_{PDEcamp}V_{AC}}{{\bar{v}}_{PDE}(\alpha_{PDE}+\beta_{PDE}\frac{{C_{iss}}^{3}}{{C_{iss}}^{3}+K_{PDEca}^{3}})-V_{AC}}$$ (12)

where *V*_*AC*_ is the rate of adenylyl cyclase production and is inhibited by *AMP*_*c*_ (Eq 2). *AMP*_*c*_ changes slowly during a burst (Fig 8A, blue) due to the activity of the glycolytic oscillator. The steady-state cytosolic Ca^2+^ concentration in Eq 12 (*c*_*iss*_) is given by:

$$c_{iss}=\frac{\alpha I_{Ca}+k_{leak}c_{er}}{k_{pmca}{+k}_{leak}+k_{SERCA}}$$ (13)

where *I*_*Ca*_ is a function of *V* and *c*_*er*_ is clamped at its mean value. This gives the voltage dependence to the *c*_*∞*_ curve.

During the burst, the glycolytic oscillator moves the *c*_*∞*_ curve back and forth. In Fig 8D the curve is plotted for values of *AMP*_*c*_ at its minimum and its maximum during a burst. During a burst *AMP*_*c*_ moves between these minimum and maximum values and shifts the *c*_*∞*_ curve back and forth. For small values of *AMP*_*c*_, the *c*_*∞*_ curve is shifted to the right (magenta dashed curve), intersecting the z-curve on the bottom stationary branch. At this point the system is in its hyperpolarized silent phase. As AMP_c_ slowly increases the *c*_*∞*_ curve shifts to the left and the phase point follows it. When the curve passes the knee, the phase point is attracted to the periodic spiking branch, starting the active phase. The phase point follows the periodic branch to the left until *AMP*_*c*_ reaches its maximum (green dashed curve). From here *AMP*_*c*_ declines and shifts the *c*_*∞*_ curve rightward, bringing the phase point with it. The *c*_*∞*_ curve eventually reaches SN2 again and intersects the stable stationary branch initiating a silent phase. It keeps moving rightward as *AMP*_*c*_ continues to decline, bringing the phase point with it. Eventually *AMP*_*c*_ begins to rise, restarting the cycle. This is parabolic bursting since the spike frequency during a burst follows a parabolic time course, low at the beginning and the end as the phase point passes near the infinite-period SNIC bifurcation [53]. As the fast subsystem bifurcation diagram lacks a bistable region, the glycolytic oscillations are necessary for the production of bursting in the Kir2.1 model.

### An alternate bursting mechanism

**Fig 9:**
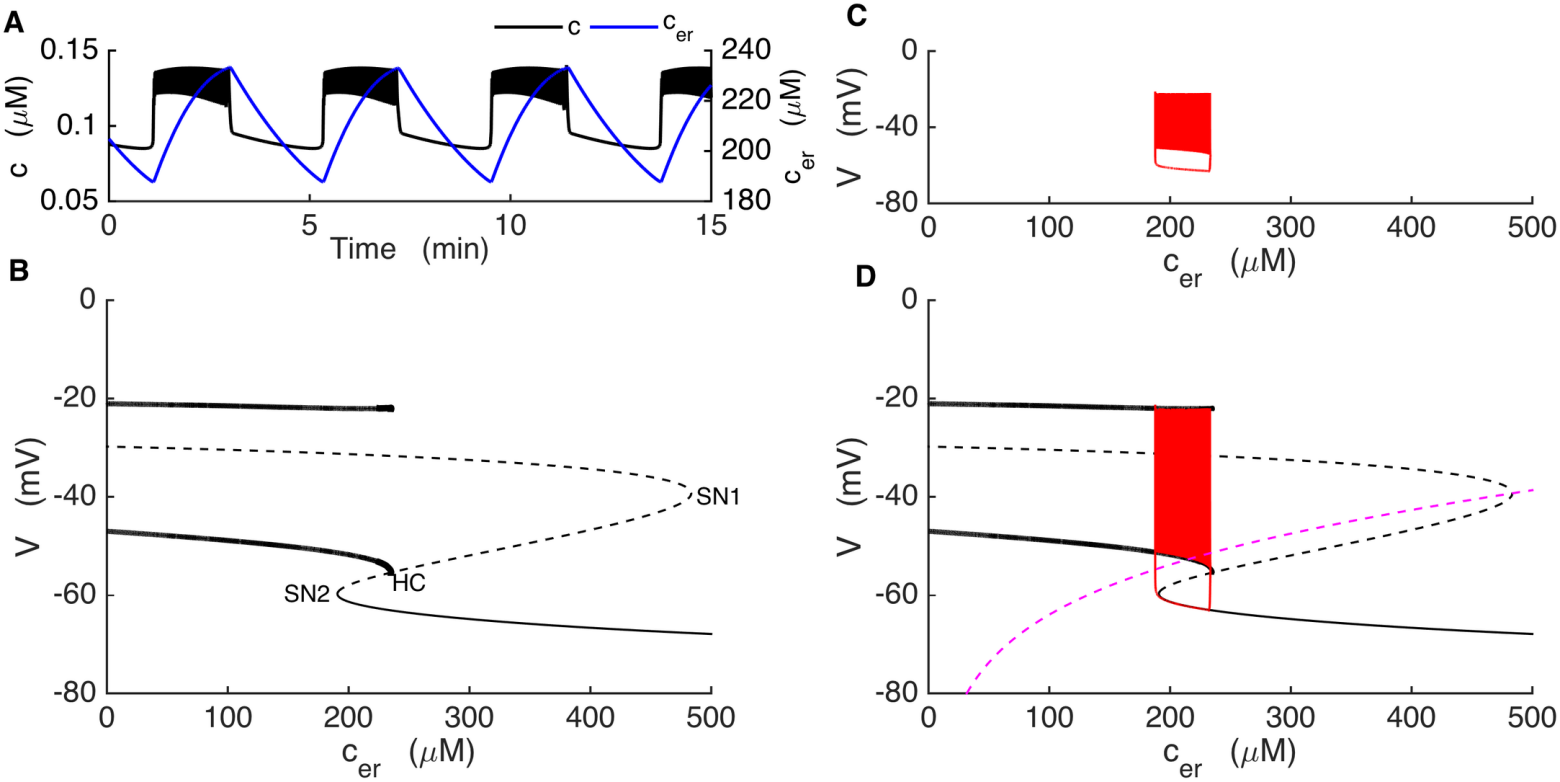
The model KO cell can produce bursting with upregulation of a constant-conductance (leak) K^+^ current and a K(Ca) conductance: *g*_*leak*_ = 32.5 pS, *g*_*K*(*Ca*)_ = 90 pS. (A) Negative feedback of *c* (black) on the membrane potential and slow *c*_*er*_ (blue) oscillations drive bursting. (B) The fast-subsystem bifurcation diagram exhibits an interval of bistability between the saddle-node bifurcation SN2 and the homoclinic bifurcation HC. (C) A projection of the burst trajectory. (D) Fast/slow analysis, with the burst trajectory (red) and the *c*_*er*_ nullcline (magenta) superimposed on the fast-subsystem bifurcation diagram. The trajectory moves leftward during the silent phase and rightward during the active phase.

To address whether the upregulation of other types of K^+^ channels might yield effects similar to those of Kir2.1, we examined the effects of replacing K(ATP) current with an alternative hyperpolarizing constant-conductance or “leak” K^+^ current, instead of Kir2.1 current, and increased the K(Ca) channel conductance (Fig 9). With these modifications, bursting could be produced in the absence of K(ATP) due to Ca^2+^ feedback onto K(Ca) channels (Fig 9A). In this model, ER Ca^2+^, which played little or no role in bursting produced using the Kir2.1 model, became absolutely essential in driving the burst.

Glycolytic oscillations are now irrelevant since they do not change the membrane potential or contribute to burst generation in any way. The fast subsystem consists of three variables in this case, *V*, *n*, and *c*, and a slow variable *c*_*er*_, which we consider as a slowly-varying parameter of the fast subsystem. The fast-subsystem bifurcation diagram is shown in Fig 9B. Unlike with the Kir2.1 model (Fig 8), there is a bistable interval in the z-curve, where stable steady states coexist with stable periodic solutions (between the saddle-node bifurcation SN2 and the homoclinic bifurcation HC). The burst trajectory is projected into the *c*_*er*_*-V* plane in Fig 9C, and superimposed on the fast-subsystem bifurcation diagram in Fig 9D. Also superimposed is the *c*_*er*_ nullcline, the curve where the *c*_*er*_ derivative is 0. Bursting is produced as the trajectory moves to the left along the bottom stationary branch of the z-curve during the silent phase and to the right along the periodic branch during the active phase, utilizing the fast-subsystem bistability. This is standard square-wave or type 1 bursting that has been described previously for other models of bursting in β-cells and in neurons [52,54].

### Experimental test distinguishes the two models

We have thus far described two possible ways in which the upregulation of hyperpolarizing K^+^ channels can rescue bursting in SUR1^-/-^ β-cells. As one clear difference between the two alternative mechanisms is their dependence on ER Ca^2+^ concentration, we explored the consequences of manipulating the ER Ca^2+^ concentration as a way of determining which model is more likely the correct one. This can be done experimentally by blocking the Ca^2+^ pumps on the ER membrane (the SERCA pumps) using the agent thapsigargin [55].

**Fig 10:**
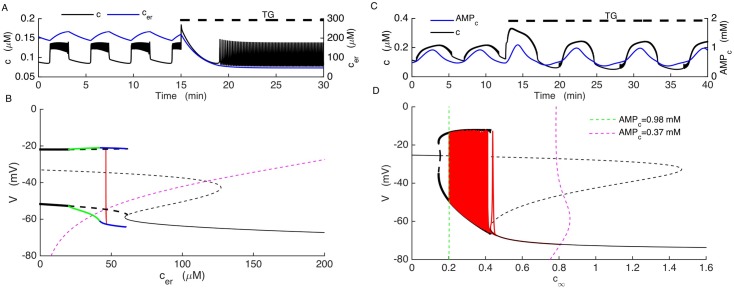
Distinct model predictions of the effects of partial inhibition of SERCA pumps with thapsigargin distinguishes the two models. (A) In the model where bursting is driven by oscillations in the ER calcium concentration simulation of TG application reduces the *c*_*er*_ (red) and terminates slow *c* oscillations (black). (B) In this model, the z-curve and *c*_*er*_ nullcline are shifted far to the left and the periodic spiking branch is destabilized. The new stable periodic branch exhibits fast two-spike bursting at the value of *c*_*er*_ at which the trajectory settles. (C) In the model in which bursting is driven by oscillations in the Kir2.1 current, bursting continues after TG application (black) because the *AMP*_*c*_ oscillations (red) persist. (D) In this model, TG increases the amplitude of the *AMP*_*c*_ oscillations, which shifts the *c*_*∞*_ curve further to the right and increases the period of oscillations, but the burst mechanism is unaltered.

In the model, the parameter *k*_*SERCA*_ is the Ca^2+^ pumping rate into the ER from the cytosol. To mimic the effect of thapsigargin we reduced *k*_*SERCA*_ by a factor of 4. In the ER bursting model, this greatly lowered *c*_*er*_ (Fig 10A, blue trace) and converted slow bursting into fast two-spike bursting (Fig 10A, black trace). In terms of the fast/slow analysis (Fig 9B), the reduction in *k*_*SERCA*_ shifts the z-curve and *c*_*er*_ nullcline far to the left. In addition, the periodic tonic spiking branch is destabilized through a period doubling bifurcation, and the resulting period doubled branch itself loses stability at a period doubling bifurcation. In fact, there is a period doubling cascade (green curve), leading ultimately to a branch of fast two-spike bursting (blue curve). The trajectory (red curve) moves to this latter curve at the new equilibrium value of *c*_*er*_. Thus, the slow bursting is replaced by very fast 2-spike bursting.

In the Kir2.1 model, in contrast, bursting persisted even when SERCA pumps were inhibited (Fig 10C, black). This is because bursting in this case is driven by the activity of the glycolytic oscillator. Blocking SERCA pumps lowers mean *c*_*er*_, which affects the cytosolic Ca^2+^ level, but this only modulates the slow bursting pattern rather than abolishing it. Indeed, the fast/slow analysis illustrates that the burst mechanism is very similar in this case to what it was before the reduction in *k*_*SERCA*_ (Fig 10D). The main difference is that the period of bursting is now increased, since the *c*_*∞*_ curve moves further to the right during the silent phase (Fig 10D, dashed magenta curve).

**Fig 11:**
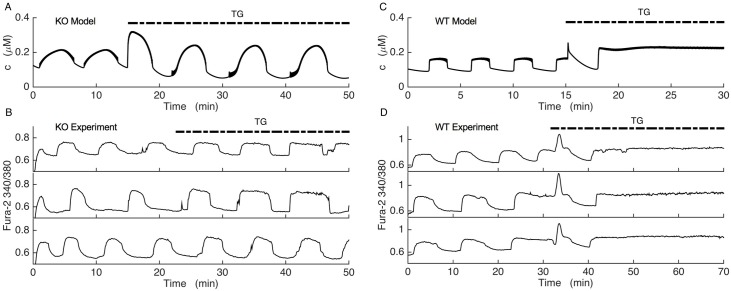
Fura-2 Ca^2+^ measurements of SUR1^-/-^ and wild-type islets compared with model simulations. In the experiments, the change in Ca^2+^ is expressed as the fura-2 340/380 fluorescence ratio. (A) In the Kir2.1 model, the parameter *k*_*SERCA*_ is reduced by a factor of 4 to mimic application of the SERCA pump blocker thapsigargin (TG). (B) Fura-2 Ca^2+^ measurements from 3 representative SUR1^-/-^ islets. Islets were maintained in 11 mM glucose, and the irreversible SERCA pump blocker TG was applied as indicated. Slow Ca^2+^ oscillations persisted after TG application in all 10 KO islets tested, as predicted by the model. (C) In the wild-type model, parameter *k*_*SERCA*_ was reduced by a factor of 4 to simulate TG application. D) Fura-2 Ca^2+^ measurements from 3 representative wild-type islets maintained in 11 mM glucose. TG was applied as indicated. Slow Ca^2+^ oscillations were replaced by sustained elevation in Ca^2+^ reflecting continuous spiking or fast bursting in 13 of 14 wild-type islets tested, as predicted.

These simulations make a testable prediction that can eliminate one or the other of the compensation models. We subsequently tested the predictions in the lab, by treating oscillating SUR1^-/-^ islets with thapsigargin (TG). Fig 11 shows the model prediction obtained with the Kir2.1 model on the top row and the results of the experiments on the bottom three rows (three SUR^-/-^ islets and three wild-type islets are shown). TG application did not terminate slow Ca^2+^ oscillations in any of the SUR1^-/-^ islets shown (Fig 11B), as predicted by the Kir2.1 model (Fig 11A). In fact, Ca^2+^ oscillations persisted in all 10 of the KO islets tested, with only a small change in the properties of the oscillations. Before TG treatment the oscillation period was 7.3 ± 1.2 min and the duty cycle (duration of elevated Ca^2+^ divided by the period) was 0.4 ± 0.08. After TG application there was a slight increase in period to 7.6 ± 1.1 min and the duty cycle increased to 0.6 ± 0.06. The slow Ca^2+^ decline that occurs at the end of each active phase prior to TG application, characteristic of Ca^2+^ leaking out of the ER and into the cytosol, was eliminated by the application of TG, as expected [56,57]. The persistence of oscillations when TG is applied is in clear contrast with the wild-type model (the model that has K(ATP) current) and wild-type islets, where in most of the wild-type islets tested TG converted slow oscillations (with period 10.6 ± 0.9 min and duty cycle 0.5 ± 0.06) to continuous spiking or fast bursting with an elevated cytosolic Ca^2+^ level (in 13 of 14 islets tested) (Fig 11C and 11D). A similar effect of TG on slow Ca^2+^ oscillations was previously observed in islets [58]. Since the response to TG confirms the prediction of the Kir2.1 model, but not the ER bursting model, we conclude that the Kir2.1 model is a more likely candidate to account for the compensation that occurs in SUR1^-/-^ islets. That is, the data support the hypothesis that bursting observed in KO islets is due to compensatory upregulation of Kir2.1 channels.

## Discussion

The primary aim of this modeling study was to help understand how islet β-cells can compensate for the genetic knockout of K(ATP) channels in SUR1^-/-^ mice. One focus was on Kir2.1 channels, which we found to be upregulated in the SUR1^-/-^ mice (Vadrevu et al, manuscript in preparation). We showed that upregulation of these channels can maintain bursting, even though the K(ATP) channels that normally couple metabolic oscillations to plasma membrane K^+^ channel activity are missing. This requires that the Kir channels have a dependence on cAMP, as has been reported previously for Kir2.1 channels [38–41]. It has also been reported that cAMP exhibits slow oscillations in insulin-secreting MIN6 cells [43] and in islet β-cells [42,43], a behavior which could reflect oscillations in the nucleotide AMP [44]. Indeed, we were not able to observe bursting in simulations of K(ATP) KO islets if AMP regulation of cAMP was omitted. We did, however, show an alternative mechanism that could produce bursting in the KO islets in a manner that is independent of Kir2.1 current. The two models made very different predictions for the effects of blocking Ca^2+^ pumps in the ER membrane, and subsequent experiments with the SERCA pump blocker thapsigargin supported the Kir2.1 model over the alternate model. Of course, we do not suggest that these are the only two models that might be capable of mediating bursting in the absence of K(ATP). For example, there are data showing that Mg:ATP can stimulate Kir channels, providing another means by which metabolic oscillations could cause bursting electrical activity [38]. We do show, however, that the two models examined herein are both feasible, and that they are experimentally discernable.

A key hypothesis that we make in the Kir2.1 model is that cAMP regulates Kir2.1 current in SUR1^-/-^ islets, likely through PKA as described in [38–41], rather than direct metabolic regulation of the channels. A consequence of this hypothesis is that manipulations that increase the cAMP level should hyperpolarize the islet and terminate Ca^2+^ oscillations. Indeed, we found this to be the case. Application of 8-Br-cAMP had no apparent effect on Ca^2+^ oscillations in wild-type islets (Fig 7A), but terminated oscillations and brought Ca^2+^ to a resting level in SUR1^-/-^ islets. This is what we predict, since we expect little or no expression of Kir2.1 channels in wild-type islets, but significant expression in SUR1^-/-^ islets (Fig 3). The data of Fig 7 do not preclude the possibility that cAMP activates another type of K^+^ channel in SUR1^-/-^ islets, but other data show that the upregulated current is an inward-rectifying K^+^ current (Fig 3). If this upregulated Kir current were regulated directly by metabolism rather than cAMP, it is hard to explain why increasing the cAMP level with membrane permeable 8-Br-cAMP would terminate Ca^2+^ oscillations and bring Ca^2+^ to a resting level.

Another hypothesis that we make is that cAMP oscillates in SUR1^-/-^ islets. This has not yet been demonstrated, as it has been in wild-type islets [42,43]. However, we have previously reported that slow NAD(P)H oscillations persist in the SUR1^-/-^ islets (Merrins et al, 2010), indicating the existence of metabolic oscillations which could drive cAMP oscillations as in our model. In glucose-stimulated wild-type islets the glycolytic product fructose 1,6-bisphosphate exhibits oscillations coincident with electrical bursting and Ca^2+^ oscillations [59], and there are slow oscillations in oxygen consumption [60] and NAD(P)H [61,62]. At present, we do not yet know if the metabolic oscillations in fact result in cyclic AMP oscillations in SUR1^-/-^ islets.

The upregulation of Kir2.1 channels we propose might result from the expected increase in β-cell electrical activity that occurs when K(ATP) channel formation is disrupted by the genetic deletion of SUR1, although when this occurs developmentally is not clear. It is well established that dramatic changes in electrical activity can regulate the expression of ion channels in excitable cells [63–65]. This may result from the increased intracellular Ca^2+^ concentration that accompanies increased electrical activity, which can enhance gene expression [66,67]. This feedback process would guard against the production of excessive Ca^2+^ levels in the cell, which can in turn induce apoptosis [68].

One prediction of the Kir2.1 model is that the Ca^2+^ and cAMP oscillations should be 180° out of phase with one another in the KO cells (Fig 5A). This differs considerably from the wild-type case, where cAMP has a saw-tooth pattern and declines during the burst active phase and then rises during the silent phase (Fig 4A). While cAMP levels have been measured simultaneously with Ca^2+^ in MIN6 cells and the time course is in general agreement with the model [43], such measurements have not yet been made in SUR1^-/-^ islets. A study performed in MIN6 β-cells in which Ca^2+^ oscillations were induced with the aid of the K^+^ channel blocker tetraethylammonium (TEA) showed oscillations in protein kinase A activity that was generally in phase with cAMP oscillations, indicating that the kinase kinetics were sufficiently fast to resolve the roughly 6-min oscillations in the cAMP concentration [69]. Our model would predict this, for both wild-type and SUR1^-/-^ islets. The PKA oscillations could affect islet β-cells in ways other than or in addition to phosphorylation of Kir channels, such as phosphorylation of L-type Ca^2+^ channels as has been demonstrated in the TC3 β-cell line [70].

Glycolytic oscillations are well established in yeast [71], but until recently there was no direct evidence that they occur in islet β-cells. However, recent studies using a FRET sensor for the glycolytic enzyme pyruvate kinase provide direct evidence for the existence of glycolytic oscillations in islets [72,59]. These metabolic oscillations are readily transmitted to the membrane potential through the cyclic activity of K(ATP) channels [46](Fig 4), and we have now illustrated how these can drive bursting even in the absence of K(ATP) channels by utilizing the cAMP dependence of upregulated Kir2.1 channels. It is not obvious how Kir2.1 channel expression increases to an appropriate level so that bursting is produced when K(ATP) channels are missing, but it is plausible that channel compensation is achieved through the actions of Ca^2+^ on Ca^2+^-dependent activators or inhibitors of transcription factors. A further modeling study for the dynamic regulation of Kir2.1 channel expression is currently under way.

## Supporting information

S1 Text Model equations and parameters. Equations and parameter values for the model.
(DOCX)

S1 Data Data for Fig 3A, calcium oscillations in a wild-type islet. (TXT)

S2 Data Data for Fig 3B, calcium oscillations in a knockout islet. (TXT)

S3 Data Data for Fig 3C, I-V curves for wild-type and knockout islets. (PXP)

S4 Data Data for Fig 7A, application of 8-Br-cAMP to 11 wild-type islets. (TXT)

S5 Data Data for Fig 7B, application of 8-Br-cAMP to 9 knockout islets. (TXT)

S6 Data Data for Fig 11B, application of Tg to 10 knockout islets. (TXT)

S7 Data Data for Fig 11D, application of Tg to 14 wild-type islets. (TXT)
