# Supplementary material for: Upregulation of an inward rectifying K+ channel can rescue slow Ca2+ oscillations in K(ATP) channel deficient pancreatic islets
Source: PLoS Comput Biol. 2017 Jul 27;13(7):e1005686. doi: 10.1371/journal.pcbi.1005686 (PMC5549769; doi:10.1371/journal.pcbi.1005686)
Supplement: S1 Text — Equations and parameter values for the model. (DOCX) [file pcbi.1005686.s001.docx]

**Supporting information**

**S1 Appendix**

We use an 8-variable model of ordinary differential equations consisting of several modules, described below.

**The Glycolytic and Mitochondrial Model**

The glycolytic model explains the dynamics of the metabolic oscillations, which result from the allosteric activity of the enzyme phosphofructokinase (PFK) (for details see [1]). A complete mathematical and biophysical description of the model can be found in [2–4]. In the model the glycolytic output is given by the output of the PFK reaction, which phosphorylates fructose 6-phosphate (F6P) to fructose 1,6-bisphosphate (FBP). FBP is then assumed to be proportional to the substrate concentration for the enzyme pyruvate dehydrogenase (PDH), described by the following flux rate:

$J_{PDH}=k_{PDH}\left( \frac{c_{m}}{c_{m}+k_{PDHcm}} \right)\sqrt{FBP/1\mu M}$ (A1)

where *k_PDH_* is the rate constant, $c_{m}=5c$ is mitochondrial Ca^2+^ concentration [5]. The rate of change of F6P and FBP concentrations are given by:

$\frac{dF6P}{dt}=k_{F6P}\left( J_{GK}-J_{PFK} \right)$ (A2)

$\frac{dFBP}{dt}=k_{FBP}\left( J_{PFK}-0.5J_{PDH} \right)$ (A3)

where *k_F6P_* and *k_FBP_* are the proportionality constants derived from a series of reactions upstream and downstream to PFK (for derivation see [4]), and *J_GK_* is the constant flux through glucokinase. This rate would be determined by factors such as the glucose concentration, the expression and the activity of the glucose transporters (GLUT2), and the expression of the glucokinase enzyme itself. The different *J_GK_* values used in the model wild-type and KO cell simulations may be regarded as a result of altered gene expression.

The mitochondrial metabolism model is adopted from [3]. It is assumed that the sum of the mitochondrial ADP (*ADP_m_*) and ATP (*ATP_m_*) concentrations is constant: *A_mtot_= ADP_m_+ ATP_m_*. *ADP_m_* is in rapid equilibrium with *J_PDH_* according to:

${ADP}_{m}=\frac{k_{ADPm}}{exp(\frac{J_{PDH}}{k_{ADPmPDHP}})}$ . (A4)

The ATP produced in the mitochondria enters the cytosol via the adenine nucleotide translocator (ANT) with flux rate:

$J_{ANT}=\frac{v_{ANT}}{1+k_{ANT}\frac{{ADP}_{m}}{{ATP}_{m}}}\exp\left( {\frac{\rho}{2}\psi}_{m} \right)$ (A5)

where $\rho=\frac{F}{RT}$ is Faraday’s constant divided by the gas constant and temperature, and $\psi_{m}$ is the mitochondrial membrane potential. The rate of change of the cytosolic ATP concentration, *ATP_c_,* is determined by ATP translocation and hydrolysis:

$\frac{d{ATP}_{c}}{dt}=V_{mc}J_{ANT}-J_{hyd}$ (A6)

where *V_mc_* is the ratio of the volume of the mitochondria to the volume of the cytosol. $J_{hyd}$ is the ATP hydrolysis due to cellular activity, given by:

$J_{hyd}=\left( k_{hyd}c+J_{hyd,bas} \right){ATP}_{c}$ (A7)

where the first term represents the ATP hydrolysis due to the fueling of Ca^2+^ pumps on the plasma and endoplasmic reticulum membranes and depends on *c,* and the second term is ATP utilization for other processes. Cytosolic AMP and ADP concentrations (*AMP_c_* and *ADP_c_,* respectively) are in rapid equilibrium with ATP and are given as in [6]:

${ADP}_{c}=\frac{{ATP}_{c}}{2K_{a}}\left( \sqrt{1-4K_{a}\left( \frac{1-A_{tot}}{{ATP}_{c}} \right)}-1 \right)$ (A8)

${AMP}_{c}=K_{a}\frac{{{ADP}_{c}}^{2}}{{ATP}_{c}}$ (A9)

where *A_tot_* is the total cytosolic adenosine nucleotide concentration, assumed to be constant.

**Ca^2+^ Handling and Membrane Potential Modules**

Equations for this module of the model are adopted from [2]. The rate of change of the membrane potential is given by the following Hodgkin-Huxley type equation:

$\frac{dV}{dt}=-\left( I_{K}+I_{Ca}+I_{K\left( Ca \right)}+I_{K\left( ATP \right)} \right)/C_{m}$ (A10)

where, *C_m_* is the membrane capacitance, $I_{K}$ is the delayed rectifier K^+^ current, $I_{Ca}$ is voltage-sensitive Ca^2+^ current, $I_{K(Ca)}$ is Ca^2+^-activated K^+^ current, and $I_{K(ATP)}$ is ATP-sensitive K^+^ current.

$I_{K}=g_{K}n\left( V-V_{K} \right)$ (A11)

$I_{Ca}=g_{Ca}m_{\infty}\left( V-V_{Ca} \right)$ (A12)

$I_{K(Ca)}=g_{K(Ca)}\omega\left( V-V_{K} \right)$ (A13)

$I_{K(ATP)}=g_{K(ATP)}s_{K(ATP)}\left( V-V_{K} \right)$ (A14)

where each $g_{i}$ is the maximal conductance of the current. *n* is the activation variable of the delayed rectifier K^+^ current with dynamics given by:

$\frac{dn}{dt}=\frac{n_{\infty}-n}{\tau_{n}}$ (A15)

where $\tau_{n}$ is the time constant and the steady state function $n_{\infty}$ is given by:

$n_{\infty}=\frac{1}{1+exp(\frac{V-V_{kv}}{5})}$ . (A16)

For simplicity, we assume that the Ca^2+^ channel activation is instantaneous, described by:

$m_{\infty}=\frac{1}{1+exp(\frac{V-V_{cv}}{12})}$ . (A17)

Activation of the Ca^2+^-activated K^+^ channels is given by the variable ω, defined as:

$\omega=\frac{c^{2}}{c^{2}+K_{c}^{2}}$ (A18)

where *k_c_* is the affinity constant for Ca^2+^. The K(ATP) conductance is assumed to respond instantaneously to changes in the concentrations of the cytosolic adenine nucleotides ADP^3-^, ATP^4-^ and MgADP^-^. This relation is given by the activation function *s_K(ATP)_*, which is taken from [7]:

$s_{K\left( ATP \right)}=\frac{0.08\left( 1+\frac{2Mg{ADP}^{-}}{17mM} \right)+0.89\left( \frac{Mg{ADP}^{-}}{17mM} \right)^{2}}{\left( 1+\frac{Mg{ADP}^{-}}{17mM} \right)^{2}\left( 1+\frac{{ADP}^{3-}}{26mM}+\frac{{ATP}^{4-}}{1mM} \right)}$ (A19)

where the binding nucleotide concentrations are given as ${MgADP}^{-}=165{ADP}_{c}$*,* ${ADP}^{3-}=135{ADP}_{c}$*,* and ${ATP}^{4-}=50{ATP}_{c}$.

In the case of SUR1^-/-^ model cells the K(ATP) current is replaced by an inward rectifying K^+^ current:

$I_{Kir}=g_{Kir}k_{\infty}c_{\infty}\left( V-V_{K} \right)$ . (A20)

Here $g_{Kir}$ is the maximal Kir2.1 channel conductance, $k_{\infty}$ is voltage-dependent inactivation and $c_{\infty}$ is cAMP-dependent activation of the channels. $k_{\infty}$is described by:

$k_{\infty}=\frac{1}{1+exp\left( \frac{V-V_{kir}}{S_{kir}} \right)}$ (A21)

where *v_Kir_* is the half-activation potential and *s_Kir_* is the slope factor. cAMP-dependent and -independent components of the Kir2.1 activation are described by:

$c_{\infty}=\alpha_{camp}+\beta_{camp}\frac{{cAMP}^{4}}{{cAMP}^{4}+K_{camp}^{4}}$ (A22)

where *α_camp_* is the cAMP-independent component, and the Hill function in the second term is the cAMP-dependent component. The constant-conductance leak K^+^ current used in the ER bursting model is defined by:

$I_{K,leak}=g_{K,leak}\left( V-V_{K} \right)$ (A23)

The rate of change of free cytosolic Ca^2+^ concentration is given by:

$\frac{dc}{dt}=f_{cyt}\left( \overset{J_{mem}}{\overbrace{{-\alpha_{c}I}_{Ca}-k_{pmca}c}}+\overset{J_{ER}}{\overbrace{k_{leak}\left( c_{er}-c \right)-k_{SERCA}c}} \right)$ (A24)

where $J_{mem}$ and $J_{ER}$represent the Ca^2+^ flux across the plasma membrane and net flux out of the endoplasmic reticulum (ER), respectively. Here, *f_cyt_* is the fraction of free to total cytosolic Ca^2+^, *α_c_* converts current to flux, *k_pmca_* is the Ca^2+^ pumping rate from the cytosol, $k_{leak}$ is the leak rate of Ca^2+^ from the ER, and $k_{SERCA}$ is the Ca^2+^ pumping rate into the ER by SERCA pumps. The ER Ca^2+^ concentration changes in time according to:

$\frac{dc_{er}}{dt}=-f_{er}V_{cte}\left( k_{leak}\left( c_{er}-c \right) -k_{SERCA}c \right)$ (A25)

where *f_er_* is the ratio of the concentration of free to total Ca^2+^ in the ER and *V_cte_* is the ratio of the volume of the cytosol to the volume of the ER compartment.

Equations for the dynamics of cAMP are adopted from [6], where the cAMP concentration is given by the difference between its production by adenylyl cyclase (*V_AC_*) and degradation by phosphodiesterases (*V_PDE_*):

$\frac{dcAMP}{dt}=V_{AC}-V_{PDE}$ (A26)

where,

$V_{AC}=\bar{v}_{AC}\left( \alpha_{AC}+\beta_{AC}\frac{c^{3}}{c^{3}+K_{ACca}^{3}} \right)\left( \beta_{amp}\frac{K_{ACamp}^{2}}{{AMP}_{c}^{2}+K_{ACamp}^{2}} \right)$ (A27)

$V_{PDE}=\bar{v}_{PDE}\left( \alpha_{PDE}+\beta_{PDE}\frac{c^{3}}{c^{3}+K_{PDEca}^{3}} \right)\frac{cAMP}{cAMP+K_{PDEcamp}}$ . (A28)

The free cytosolic Ca^2+^ stimulates both production and degradation of cAMP by acting on adenylyl cyclase and phosphodiesterases with different affinities, $K_{ACca}$ and $K_{PDEca}$, respectively. AMP inhibits the cAMP production by inhibiting adenylyl cyclase. $\alpha_{AC}$ and $\alpha_{PDE}$ are basal cAMP production and degradation rates, respectively. All model parameter values are given in Table S1.

| Table S1: Parameter Values | | | |
| --- | --- | --- | --- |
| $k_{PDH}$ | 0.00037 μMms^-1^ | $k_{leak}$ | 4.14x10^-5^ ms^-1^ |
| $k_{PDHcm}$ | 0.1 μM | $V_{k}$ | -75 mV |
| $J_{GK}$ | 0.09 or 0.14μMms^-1^ | $g_{K}$ | 486 pS |
| $k_{F6P}$ | 0.136 | $v_{kv}$ | -16 mV |
| $k_{FBP}$ | 0.8 or 0.95 | $\tau_{n}$ | 1000/9 ms |
| $A_{mtot}$ | 15 mM | $g_{K(Ca)}$ | 18 pS |
| $k_{ADPm}$ | 12.5 | $k_{c}$ | 0.5 μM |
| $v_{ANT}$ | 0.00007245 μMms^-1^ | $g_{K(ATP)}$ | 2960 pS |
| $k_{ANT}$ | 2 | $K_{ACca}$ | 0.08 μM |
| $\rho$ | 0.037 mV^-1^ | $\beta_{AC}$ | 3 |
| $A_{ctot}$ | 2.5 mM | $\alpha_{AC}$ | 0.5 |
| $K_{a}$ | 0.8 | $K_{PDEca}$ | 0.1 μM |
| $\psi_{m}$ | 164 mV | $\bar{v}_{AC}$ | 0.00018 μMms^-1^ |
| $V_{mc}$ | 39/532 | $\bar{v}_{PDE}$ | 0.0024 μMms^-1^ |
| $k_{hyd}$ | 0.0000234 μM^-1^ms^-1^ | $\alpha_{PDE}$ | 0.4 |
| $J_{hyd,bas}$ | 0.0000081 ms^-1^ | $\beta_{PDE}$ | 1.2 |
| $C_{m}$ | 5300 fF | $k_{PDEcamp}$ | 3 μM |
| $f_{cyt}$ | 0.0086 | $k_{ACamp}$ | 0.2 mM |
| $V_{Ca}$ | 25 mV | $\beta_{amp}$ | 1.9 |
| $g_{Ca}$ | 180 pS | $K_{camp}$ | 0.22 μM |
| $v_{cv}$ | -20 mV | $\alpha_{camp}$ | 0.2 |
| $\alpha_{c}$ | 5.17x10^-6^ fA^-1^μMms^-1^ | $\beta_{camp}$ | 0.8 |
| $k_{pmca}$ | 0.0414 ms^-1^ | $g_{Kir}$ | 110 pS |
| $V_{cte}$ | 620/23 | $v_{kir}$ | -49 mV |
| $f_{er}$ | 0.01 | $s_{kir}$ | 15 mV |
| $k_{SERCA}$ | 0.0828 ms^-1^ | $k_{ADPmPDH}$ | 0.0047 mM/s |

1. Smolen P. A model for glycolytic oscillations based on skeletal muscle phosphofructokinase kinetics. J Theor Biol. 1995;174: 137–148. doi:10.1006/jtbi.1995.0087

2. Bertram R, Satin L, Zhang M, Smolen P, Sherman A. Calcium and glycolysis mediate multiple bursting modes in pancreatic islets. Biophys J. 2004;87: 3074–87. doi:10.1529/biophysj.104.049262

3. Watts M, Fendler B, Merrins M, Satin LS, Bertram R, Sherman A. Calcium and metabolic oscillations in pancreatic islets: Who’s driving the bus? SIAM J Appl Dyn Syst. 2014;13: 683–703. doi:10.1137/130920198.

4. McKenna JP, Ha J, Merrins MJ, Satin LS, Sherman A, Bertram R. Ca^2+^ effects on ATP production and consumption have regulatory roles on oscillatory islet activity. Biophys J. 2016;110: 733–742. doi:10.1016/j.bpj.2015.11.3526

5. Merrins MJ, Poudel C, McKenna JP, Ha J, Sherman A, Bertram R, et al. Phase analysis of metabolic oscillations and membrane potential in pancreatic Islet β-cells. Biophys J. 2016;110: 691–699. doi:10.1016/j.bpj.2015.12.029

6. Peercy BE, Sherman AS, Bertram R. Modeling of glucose-induced cAMP oscillations in pancreatic β-cells: cAMP rocks when metabolism rolls. Biophys J. 2015;109: 439–449. doi:10.1016/j.bpj.2015.06.024

7. Magnus G, Keizer J. Model of β-cell mitochondrial calcium handling and electrical activity. I. Cytoplasmic variables. Am J Physiol. 1998;274: C1158–C1173.
